# Clinical recovery in children with uncomplicated appendicitis undergoing non-operative treatment: secondary analysis of a prospective cohort study

**DOI:** 10.1007/s00431-018-3277-9

**Published:** 2018-11-12

**Authors:** Max Knaapen, Johanna H. van der Lee, Hugo A. Heij, Ernst L. W. van Heurn, Roel Bakx, Ramon R. Gorter

**Affiliations:** 1 Pediatric Surgical Center of Amsterdam, Emma Children’s Hospital and VU University Medical Center, Amsterdam UMC, University of Amsterdam, P.O. Box 22660, 1100 DD Amsterdam, The Netherlands; 2 Pediatric Clinical Research Office, Academic Medical Center, Emma Children’s Hospital and VU University Medical Center, Amsterdam UMC, University of Amsterdam, Meibergdreef 9, 1105 AZ Amsterdam, The Netherlands

**Keywords:** Pediatric surgery, Non-operative treatment, Uncomplicated appendicitis, Clinical recovery

## Abstract

Non-operative treatment of uncomplicated appendicitis in children is gaining ground. Pending definitive evidence regarding its effectiveness, there is a call to evaluate clinical recovery after non-operative treatment. In this study, we analyzed data collected during initial admission of a multicenter prospective cohort in which children, 7–17 year, were treated non-operatively for uncomplicated appendicitis. During admission clinical parameters (pain and gastro-intestinal symptoms), inflammation parameters and sequential abdominal ultrasound were recorded. In total, 45 children were included, 42(93%) were discharged without the need for appendectomy; median [IQR] pain scores on admission were 5 [4–7], decreasing to 2 [0–3] after 1 day of treatment. Initially, 28/42 (67%) reported nausea and 19/42 (45%) vomiting; after 1 day, this was 3/42 (7%) and 1/42 (2%), respectively. White blood cell count declined from a median [IQR] of 12.9 [10.7–16.7] 10E9/L on admission to 7.0 [5.8–9.9] 10E9/L on day 1. Median [IQR] C-reactive protein levels *increased* from 27.5 [9–69] mg/L on admission to 48 [22–80] mg/L on day 1, declining to 21.5 [11–42] mg/L on day 2. Follow-up ultrasound showed no signs of complicated appendicitis in any of the patients.

*Conclusion*: Clinical symptoms resolved in most children after 1 day of non-operative treatment. This suggests that non-operative treatment is a viable alternative to appendectomy regarding clinical recovery.

*Trail registration*: NCT01356641

**Table:** 

**What is Known:** • *Non-operative treatment of uncomplicated appendicitis in children is safe and its use around the world is gaining ground, however high quality evidence from adequately designed randomized trials is still lacking.* • *Concerns have been raised regarding the potentially prolonged clinical recovery associated with non-operative treatment*.	
**What is New:** • *Most clinical symptoms resolve after 1 day of non-operative treatment in the majority of children*.

**Electronic supplementary material:**

The online version of this article (10.1007/s00431-018-3277-9) contains supplementary material, which is available to authorized users.

## Introduction

Appendicitis is a common gastro-intestinal disease affecting approximately 1 per 1000 inhabitants each year in the Western world [2, 4], with the highest incidence between the ages of 10 and 19 years. Historically, the standard treatment for acute appendicitis is appendectomy. This is based upon the assumption that appendicitis is an irreversible progressive disease, leading to perforation of the appendix with subsequent peritonitis. Removal of the inflamed appendix as soon as possible when appendicitis was suspected was therefore considered necessary. Nowadays, the progressive nature of appendicitis is being questioned, indicating two types of appendicitis: uncomplicated (without tendency for perforation) and complicated (with tendency to perforate) [5, 9, 24, 36]. Therefore, the necessity of an appendectomy is being challenged and non-operative treatment (NOT) of uncomplicated appendicitis in children is gaining ground. Recently five systematic reviews [13, 15, 19, 21, 42] were published, including 13 studies with a total of 808 children treated non-operatively. The included study designs comprise retrospective studies, prospective cohort studies, and one pilot randomized controlled trial (RCT). Four of these systematic reviews concluded that NOT is safe and feasible, based upon the available data. One systematic review deemed NOT inferior because of the risk of recurrent appendicitis and subsequent higher re-admission rates [21]. So far adequately powered RCTs are lacking [13, 15, 21, 42] and data on other important outcomes such as life-impact and costs is scarce. In our experience clinicians have doubts concerning the recovery of patients after NOT, particularly regarding prolonged abdominal pain and gastro-intestinal symptoms. These secondary outcomes are of value in weighing the advantages and disadvantages of NOT compared to conventional appendectomy. In contrast to patients treated surgically, who are considered to be fully recovered the following day. This call for more information on clinical recovery is also reflected in the editorial commentaries [7, 18, 25, 27] following the systematic review by Huang et al. [19]. The authors stress the need for higher-level evidence with larger pediatric patient groups and preferably randomized or patient preference designs. They also call for more data on patient-centered outcomes to determine the burden on patients and family.

One of the few repeatedly reported differences between NOT and surgical treatment is an increase in length of hospital stay (LOS) in the NOT group [13]. Reducing the length of primary admission in the NOT group could further lower the associated costs and treatment burden. In our own prospective cohort study, in which all participating children were treated non-operatively, according to protocol LOS was at least 48 h due to the intravenous (IV) administration of antibiotics and clinical monitoring [14]. However, we noticed that clinical recovery was swift in most children. Since most clinicians only have experience with operative management of appendicitis, the objective of this study is to illustrate the clinical course of patients with uncomplicated appendicitis who were successfully treated with NOT. We aim to provide a clear picture of what can be expected of non-operative management to aid clinicians in their decision-making and counseling. Secondly, we aim to provide more insight in the necessity of (prolonged) in-hospital monitoring.

## Methods

We performed the Antibiotic Versus Primary Appendectomy in Children (APAC) study, a prospective multicenter cohort pilot study (clinicaltrials.gov: NCT01356641). Patients were enrolled in one pediatric surgical center and two teaching hospitals in the Netherlands. The primary aim was to evaluate the feasibility of an RCT and the safety of NOT for uncomplicated appendicitis. The results of the first 25 patients were published in 2015 [14]. Children aged 7–17 years with a radiologically confirmed uncomplicated appendicitis were eligible for inclusion. Ultrasonography criteria included incompressible appendix with an outer diameter of > 6 mm, hyperemia within the appendiceal wall, infiltration of the surrounding fat. Excluded were children with signs of complicated appendicitis, a faecalith on imaging, signs of perforation on imaging (abscess, mass, phlegmon, disseminated peritoneal fluid, or extraluminal gas), suspicion for malignancy, severe general illness (generalized peritonitis/sepsis), severe associated conditions/malformations, or a documented type 1 allergy to the antibiotics used.

The protocol of the cohort study is described in more detail in the earlier publication [14]. To summarize, after informed consent was obtained from both children and their legal guardians, they were admitted to the (pediatric) surgical ward. Children received intravenous amoxicillin/clavulanic acid (100/10 mg/kg/day; max 6000/600 mg/day) and gentamicin (7 mg/kg/day) for at least 48 h. Repeat clinical evaluation, biochemical testing, and follow-up ultrasound were performed, starting on the next calendar day after admission. Pain was assessed using a 11-point Numeric Rating Scale (NRS) [8], with “0” representing “no pain” and “10” representing the “most severe pain imaginable.” The use of this tool for self-reporting of pain intensity is appropriate for children of 7 years and above [40]. Pain medication was prescribed according to the Dutch national guideline [11]. These measures were taken to detect possible clinical deterioration at an early stage. If after 48 h the child fulfilled predefined discharge criteria, the treatment was changed to 5 days oral amoxicillin/clavulanic acid (50/12.5 mg/kg/day). In-hospital monitoring was continued for another 24 h in the first 25 patients, whereas the subsequent 24 patients were discharged immediately with oral antibiotics. An appendectomy could be performed at any stage in case of clinical deterioration for which the predefined criteria were any of the following: increasing NRS compared to NRS on admission, signs of generalized peritonitis, temperature > 38.5 °C for more than 24 h, persistent vomiting for more than 24 h.

The medical ethics committee of the VU University medical center approved this study.

In this study, we performed an analysis of the data gathered prospectively from the previously described cohort study. The following parameters were analyzed.

- Daily clinical evaluation of: Pain levels, tenderness in right lower abdomen during palpation, nausea, vomiting.
- Daily blood samples: C-reactive protein (CRP; mg/L), white blood cell count (WBC count; 10E9/L).
- Abdominal ultrasound on admission and after 2 days of treatment.

In case of missing data, original medical records were re-examined in an attempt to retrieve the data. In case necessary data could not be retrieved from the medical records, the patient was excluded from further analysis.

### Statistical analysis

Descriptive statistics were performed using SPSS version 24.0 (IBM Corp. released 2013. Armonk, NY). Percentages are presented with their 95% confidence intervals (95%CI) between straight brackets [] calculated using the software CIA [3] (Wilson method). Non-normally distributed continuous data are presented as medians with their interquartile range (IQR) or range. Boxplots present the course of variables over time. Patients that were discharged without the need for an operation are presented separately from the patients who were operated on during primary admission.

## Results

In total, 49 patients were included in the cohort study between September 2012 and November 2015. Overall, in 5 of the 49 patients (10%), an appendectomy was performed during the first 8 weeks, 2 of the 25 patients of our initial cohort (reported earlier [14]) and 3 of the 24 patients of our subsequent cohort. Reasons to perform an appendectomy were as follows: clinical deterioration (*N* = 2), faecalith on follow-up ultrasound (*N* = 2), suspected recurrent appendicitis after 6 weeks (*N* = 1). Histopathologic examination of the suspected recurrence showed no inflammation. One of the patients with clinical deterioration was diagnosed with a perforated appendicitis during appendectomy. Within 1 month, this patient developed an intra-abdominal abscess which was treated with percutaneous drainage and antibiotics and recovered without further complications. None of the other patients suffered from complicated appendicitis or postoperative complications.

For this secondary analysis, four patients were excluded due to missing data, as shown in the CONSORT Flow diagram (Online Resource 1). General characteristics of the 45 patients included in this secondary analysis (with complete clinical data set) are shown in Table 1. Appendectomy during the clinical phase was performed in three of the 45 patients (7%) due to clinical deterioration (*N* = 2) or the detection of a faecalith on follow-up ultrasound (*N* = 1).

**Table 1:** General characteristics in/excluded patients

Variable	Included patients (*n* = 45)	Excluded patients (*n* = 4)
Age in years, median (range)	13 (7–17)	12 (8–12)
Male sex (percentage)	27 (60%)	2 (50%)
Length of hospital stay in days, median (Range)	2.5 (2.5–7.5)	2.75 (2–3)
Uneventful clinical recovery (percentage)	42 (93%)	4 (100%)

### Clinical course

#### Uneventful group (*N* = 42)

##### Pain

**Fig. 1:**
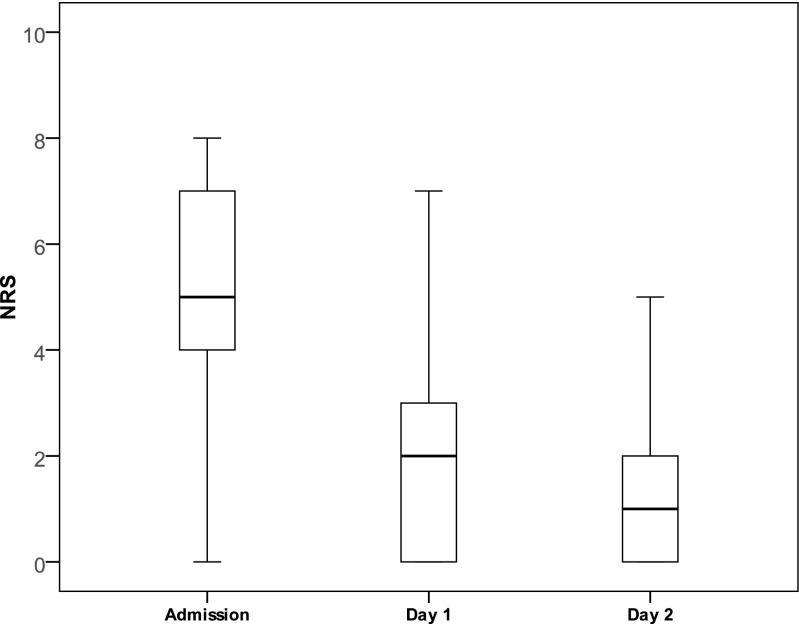
NRS. Boxplot showing NRS levels of patients with an uneventful clinical recovery (*n* = 42) on admission and after 1 and 2 days of non-operative treatment

**Fig. 2:**
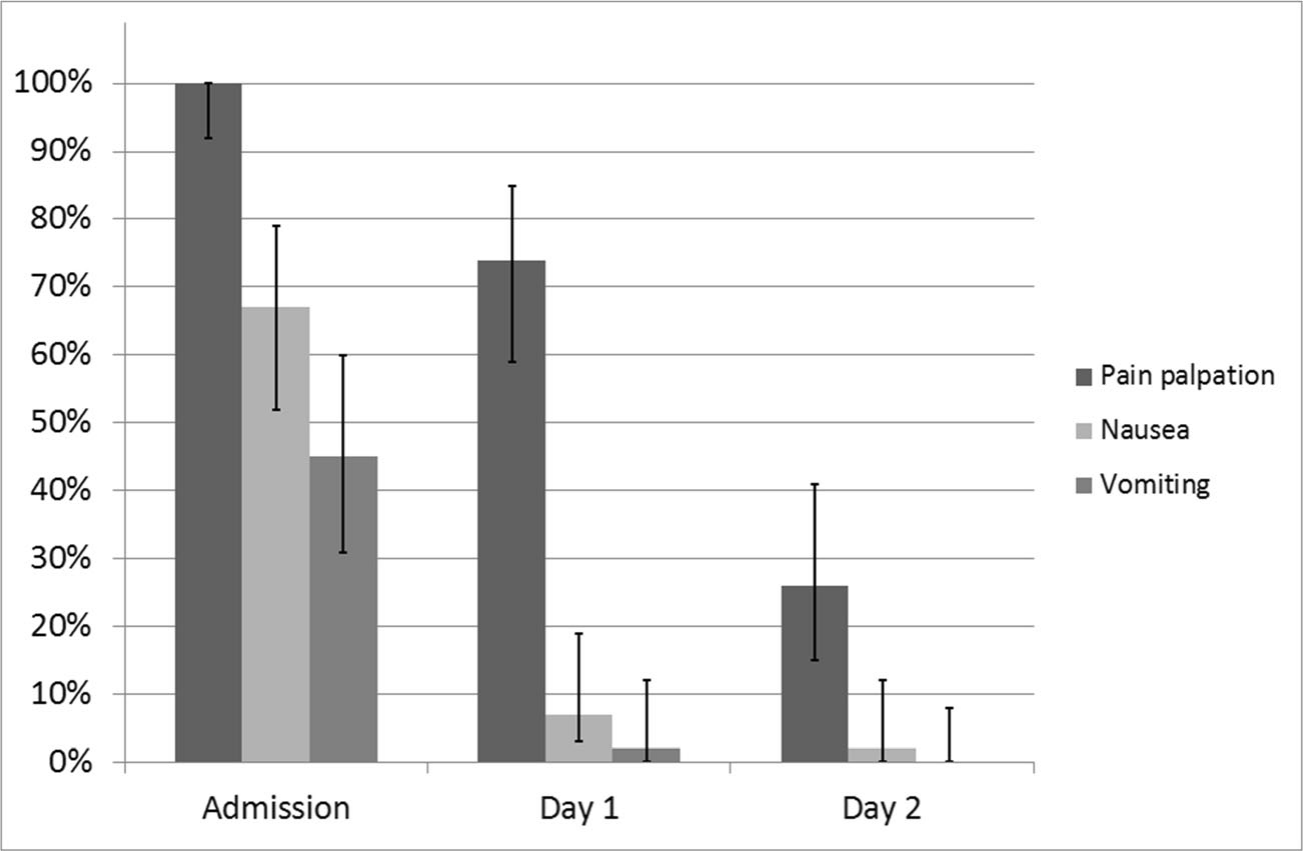
Clinical parameters. Percentages of patients with uneventful clinical recovery (*n* = 42) reporting tenderness of the right lower abdomen, nausea, or vomiting on admission and after 1 and 2 days of non-operative treatment. Presented with their 95% confidence interval

For the 42 children discharged without appendectomy, the median [IQR] NRS was 5 [4–7] on admission. This decreased to 2 [0–3] after 1 day (the next calendar day) of treatment and to 1 [0–2] on day 2. Results are shown in Fig. 1. During in-hospital stay, the presence of right lower abdomen tenderness with palpation decreased from all 42 patients (100 [92–100]%) on admission to 31/42 (74 [59–85]%) on day 1 and 11/42 (26 [15–51]%) on day 2. Results are also shown in Fig. 2.

##### Gastro-intestinal symptoms

Initially, 28/42 patients (67 [52–79]%) reported nausea and 19/42 (45 [31–60]%) vomiting. After 1 day of treatment, this was 3/42 (7% [3–19]%) and 1/42 (2 [0–12]%), respectively. On day 2, this further decreased to 1/42 (2 [0–12]%) and 0/42 (0 [0–8]%), respectively. Results are also shown in Fig. 2.

##### Parameters of inflammation

**Fig. 3:**
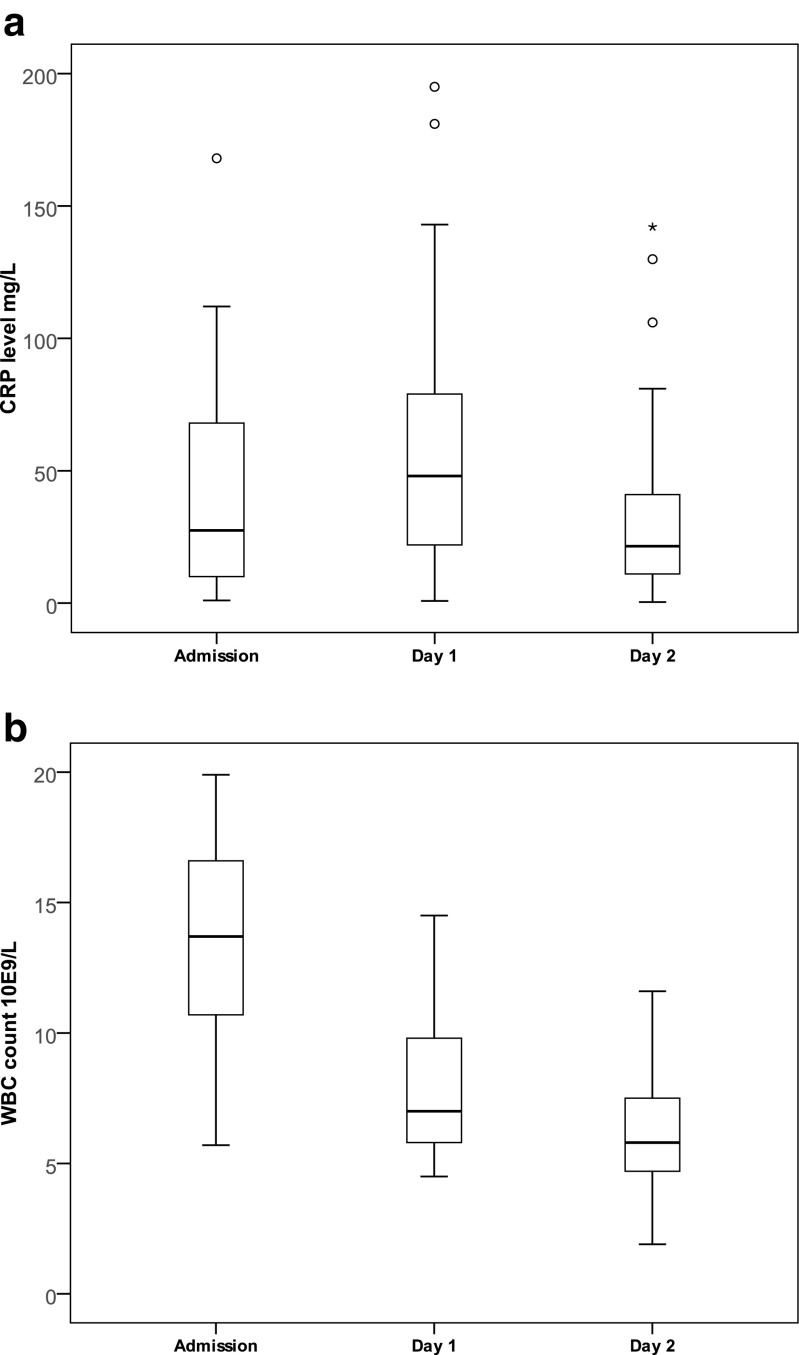
**a** Boxplot CRP levels. Boxplot showing CRP levels of patients with an uneventful clinical recovery (*n* = 42) on admission and after 1 and 2 days of non-operative treatment. **b** Boxplot WBC count. Boxplot showing WBC count of patients with an uneventful clinical recovery (*n* = 42) on admission and after 1 and 2 days of non-operative treatment

Initially, an increase in median [IQR] CRP levels was observed from 27.5 [9–69] mg/L on admission to 48 [22–80] mg/L on day 1, after which there was a decrease to 21.5 [11–42] mg/L on day 2 (Fig. 3a). In contrast to the CRP levels, median [IQR] WBC count decreased gradually during in-hospital stay from 13.7 [10.7–16.7] 10E9/L on admission, to 7.0 [5.8–9.9] 10E9/L on day 1, and to 5.8 [4.7–7.6] 10E9/L on day 2 (Fig. 3b).

##### Appendectomy group (*N* = 3)

Three patients underwent appendectomy during primary admission. Their level of pain (NRS 4, 6, and 6) was above the P75 on day 1. One patient was operated on day 1 because of clinical deterioration, with an NRS score of 6 (4 on admission) and fever (39.2 °C). The second patient in whom appendectomy was needed had a NRS score on admission of 7. On day 2, the score was 5, increasing to 6 on day 3. This was accompanied by elevated inflammation parameters. The third patient had a faecalith on repeated ultrasonography. Nausea and vomiting were absent in all three patients after 1 day. Painful palpation was present in all three patients up to the moment of appendectomy.

#### Abdominal ultrasound

All 45 patients underwent abdominal ultrasound on admission. In 41/45 patients, the appendix could be visualized. Three out of the four patients whose appendix could not be visualized underwent magnetic resonance imaging and one underwent abdominal computer tomography. Imaging confirmed the diagnosis uncomplicated appendicitis in all patients. 44/45 patients underwent follow-up ultrasound after 2 days of antibiotic treatment. One patient was operated prior to the scheduled abdominal ultrasound because of clinical deterioration. In the patient undergoing appendectomy after 3 days of treatment because of clinical deterioration, the ultrasound on day 2 showed no signs of complicated appendicitis. Also none of the other 43 patients showed signs of complicated appendicitis. In 5/42 patients with an uneventful clinical recovery, the appendix could not be visualized on follow-up ultrasound; however, in 1/5 patients, infiltration of the surrounding fat was still present. Only in 2/37 patients an appendix was visualized in which all signs of appendicitis had receded. So in 35/37 patients with an uneventful clinical recovery, the follow-up ultrasound still showed signs of appendicitis.

In three out of four patients in whom the appendix could not be visualized on admission, the appendix was also not visualized during follow-up ultrasound (the fourth patient already underwent appendectomy). No secondary signs of complicated appendicitis were seen. No other follow-up imaging was performed and these three patients were discharged uneventfully. In 2/41 patients whose appendix was visualized on admission, the appendix could not be visualized on the follow-up ultrasound. Again no secondary signs of complicated appendicitis were seen and they were discharged uneventfully.

##### Faecalith on the follow-up ultrasound

In 3/44 (7 [CI 2–18]%) patients, the ultrasound on day 2 showed a faecalith which was not seen on the initial imaging study. One of these patients underwent appendectomy during the primary admission. Histopathological examination showed a non-inflamed appendix without faecalith, with possible signs of chronic appendicitis. The two other patients with a faecalith in situ decided, in consultation with their treating surgeon, not to undergo appendectomy because of complete clinical recovery. However, both these patients underwent appendectomy within 8 weeks after discharge because of recurrent symptoms. Pathology results showed uncomplicated appendicitis with a faecalith in one and a non-inflamed appendix with fibrosis and a faecalith in the other.

## Discussion

This is the first study to describe the clinical course of children treated with non-operative treatment for uncomplicated appendicitis in detail. We show that the majority (93%) of patients who respond to antibiotic treatment recover rapidly and with only little discomfort. In the majority of children, pain was manageable and gastro-intestinal symptoms had subsided after 1 day of treatment. None of the systematic reviews in children published so far addressed the level of pain. Only one study addressed clinical recovery by reporting on abdominal pain. In 125 children who were treated non-operatively, the median period to disappearance of abdominal pain was 2.12 days [23]. Comparisons with standard appendectomy have not been made [23]. The use of analgesia is an important component of the perceived pain. Unfortunately in this study, the administered analgesics were not recorded. However, all children were treated according to the standard Dutch pediatric pain protocol [11]. In this protocol, all children were treated with oral pain medication: acetaminophen, in many cases supplemented with diclofenac. The use of morphine was very rare and only on the first day. The national protocol is also used for children undergoing appendectomy so our results should reflect the clinical recovery in routine clinical practice.

A major limitation of this study is the absence of a comparison with children undergoing appendectomy. However, the results from the first large randomized trials are expected no sooner than 2020 [12, 16, 22, 33, 41]. Thus this is currently the best available evidence regarding clinical recovery. Nonetheless, definitive answers are needed from RCTs comparing NOT to appendectomy. Also, no statistical test was performed. We decided against this because of the relatively small numbers, not normally distributed data, and the lack of a comparator group [30].

### Length of hospital stay

In most studies, length of hospital stay for children receiving NOT is longer in comparison to children undergoing immediate appendectomy. A meta-analysis including 10 studies [13] reported a mean difference [95% CI] of 0.48 [0.18–0.78] days. Despite the longer LOS associated with NOT, the reported appendicitis related healthcare costs are either reported to be lower [17, 28] or similar to that of an appendectomy [29, 37]. Decreasing the necessary length of stay could probably lead to lower medical costs and treatment burden. The discussion on the necessary length of hospital stay both after NOT and appendectomy for uncomplicated appendicitis is also reflected in the differences in study protocols. Four RCTs [12, 16, 22, 41] and one prospective patient preference study [33] are currently recruiting children with uncomplicated appendicitis to compare NOT to appendectomy, all using IV antibiotics in the non-operative arm. The minimum duration of IV antibiotic administration described in these protocols varies between 12 and 48 h. The duration of hospital admission can only be shortened if the duration of IV administration can be reduced. There is, however, no clear rationale for prolonged IV administration. In a number of other diseases, there is evidence that the duration of IV administration of amoxicillin/clavulanic acid does not influence outcomes [31, 34, 35]. In one pilot RCT in a mixed, predominantly adult population, outpatient management of appendicitis was even deemed feasible [38]. Moreover, a recent RCT in adults showed that non-operative treatment without antibiotics was non-inferior to non-operative treatment with antibiotics [32], which raises the question whether antibiotics are needed at all in the treatment of uncomplicated appendicitis. Clinical monitoring, in our opinion, is important to evaluate the response to NOT, and therefore, we would advise hospital admission for at least 24 h. Prolonged admission, however, in case of clinical improvement is questionable. As demonstrated in this study, pain decreased rapidly and fast oral intake was possible, as nausea and vomiting were no longer present after 1 day of treatment in > 90% of the children. As same-day discharge is becoming a common practice, greater time resolution of the clinical parameters would be ideal, for instance 6-h intervals. Unfortunately in this study, the clinical parameters were recorded in the morning of each calendar day. Switching to oral antibiotics and discharge after 24 h of treatment seem appropriate in patients that demonstrate an expeditious clinical recovery. This would bring the length of hospital stay for non-operative treatment close to the average length of stay in children undergoing standard appendectomy for uncomplicated appendicitis, which is 1.5 days [6, 28, 37].

### Routine biochemical and ultrasound monitoring

To detect clinical deterioration as early as possible and to evaluate the effectiveness of NOT, we incorporated routine checks of biochemical parameters and a follow-up ultrasound. In our study, in most patients, CRP levels increased in the first 24 h, after which CRP levels decreased to values similar to the levels on admission. In contrast, WBC showed a clear and immediate decline after 1 day of non-operative management. Thus, CRP levels seem useless in assessing the response to conservative treatment after 1 day. In the two patients who required appendectomy because of clinical deterioration, WBC count however also did not increase on day 1. Thus, so far, we have been unable to identify a sensitive biochemical parameter to predict clinical deterioration. Therefore, we do not recommend routine WBC or CRP analysis unless there is a clinical suspicion of deterioration or a delayed recovery. If there is clinical suspicion of deterioration, longer clinical observation or appendectomy seems warranted.

In our cohort study, abdominal ultrasound was repeated after 2 days of treatment to check for signs of complicated appendicitis. Since there was limited experience with NOT, this was done in order to maximize patient safety. In the protocol for the APAC trial [22], this same strategy is followed, as well as in several other studies [1, 10, 20]. In our cohort, the follow-up ultrasound never showed signs of complicated appendicitis. However, in 3/43 patients, a faecalith was uncovered that was not seen on the initial ultrasound. One patient was therefore operated on immediately and the other two patients underwent appendectomy within 8 weeks because of recurrent symptoms. This is in line with results from a cohort study investigating NOT in children with uncomplicated appendicitis and a faecalith on pre-operative imaging [26]. This study was discontinued early after treating five children non-operatively since three of these children suffered a recurrence at a median follow-up of 4.7 months. However, the indication for a delayed appendectomy in the case of a faecalith on follow-up ultrasound is debatable. Does this finding in a patient who has completely recovered clinically provide sufficient indication for an operation? Or can we wait and see whether the recurrence actually happens? After analyzing our data, we see no additional benefits of a standardized follow-up ultrasound to detect complicated appendicitis, as it did not lead to any changes in clinical policy in this cohort and signs of active appendicitis were still visible in most patients with an uneventful clinical recovery. Whether a second ultrasound is justified for detecting a missed faecalith because of the associated risk of recurrence is questionable. Clinical assessment combined with selective evaluation of inflammation parameters should give clinicians enough information to assess whether additional imaging is necessary or if delayed appendectomy is warranted. From these data, discharging patients without a follow-up ultrasound seems a justified strategy.

A limitation of this study is the fact that data collection was not complete for all 49 patients. However, after examination of the medical records, the amount of missing data was limited. Secondly, it is not certain whether or not patients with a non-inflamed appendix were included in this cohort study; this could influence outcomes. However, this percentage is probably low as all children underwent ultrasonography. In the Netherlands, the rate of non-inflamed appendectomy has been reduced to 3% after routine ultrasonography for all patients with possible appendicitis has been introduced [39]. Furthermore, only patients that would normally have been scheduled for an appendectomy were included in the cohort study.

Our results support the safety of NOT and illustrate the rapid recovery seen in most patients treated non-operatively. This opens the discussion and outlines the importance of conducting new studies to determine the optimal protocol with respect to the necessity of IV antibiotic administration, its duration, and the necessity (and duration) of clinical monitoring.

In conclusion, the clinical recovery of patients with uncomplicated appendicitis treated with non-operative management is fast. Clinical symptoms subside in most patients after 1 day of treatment, which makes NOT a viable alternative to appendectomy regarding clinical recovery. Nonetheless, definitive answers are needed from randomized clinical trials comparing NOT to conventional appendectomy in children with uncomplicated appendicitis. Our results also raise questions about the necessity of prolonged and intensive clinical monitoring, which is now incorporated in most study protocols investigating non-operative treatment of appendicitis. Prospective studies are warranted to investigate the possibility of earlier discharge and less stringent monitoring.

## Electronic supplementary material

ESM 1 Flowchart showing the enrollment, exclusion and follow-up of all patients with appendicitis in the participating centers during the course of the APAC cohort study. (PNG 4233 kb)

High Resolution Image (TIF 605 kb)

## Abbreviations

CRP: C-reactive protein
IV: Intravenous
IQR: Interquartile range
LOS: Length of hospital stay
NOT: Non-operative treatment
NRS: Numeric Rating Scale
RCT: Randomized controlled trial
WBC count: White blood cell count
95%CI: 95% confidence intervals
