# Supplementary figures and images for: Clinical recovery in children with uncomplicated appendicitis undergoing non-operative treatment: secondary analysis of a prospective cohort study
Source: Eur J Pediatr. 2018 Nov 12;178(2):235–42. doi: 10.1007/s00431-018-3277-9 (PMC6339679; doi:10.1007/s00431-018-3277-9)

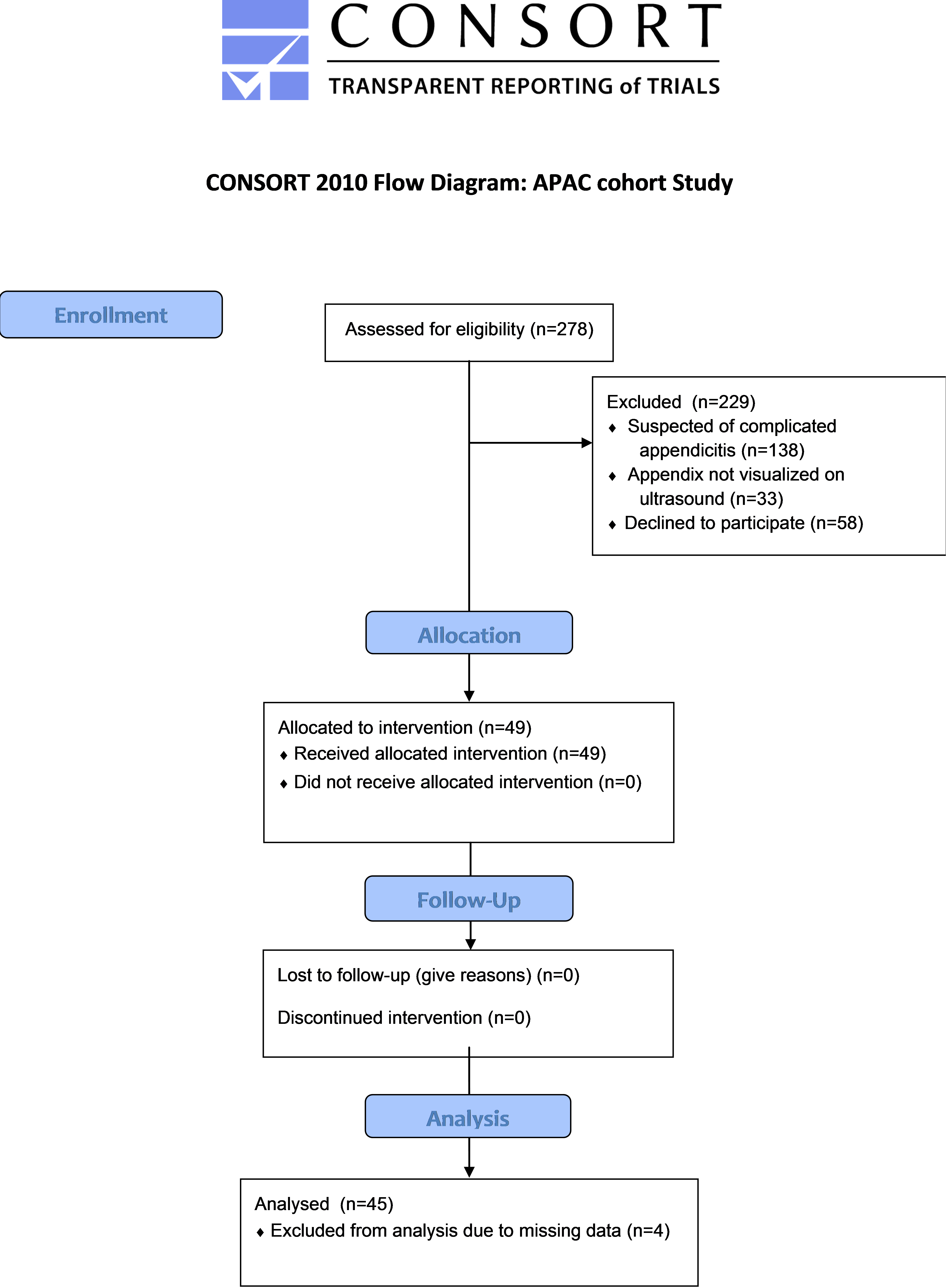

Supplement: Supplementary file 2 — High Resolution Image (TIF 605 kb) [file 431_2018_3277_MOESM1_ESM.tif]
